# Bull spermatozoa selected by thermotaxis exhibit high DNA integrity, specific head morphometry, and improve ICSI outcome

**DOI:** 10.1186/s40104-022-00810-3

**Published:** 2023-01-11

**Authors:** Sara Ruiz-Díaz, Rosane Mazzarella, Paula Navarrete-López, Raúl Fernández-González, Celia de Frutos, María Maroto, Claudia Cucala, Paula Beltrán-Breña, Marta Lombó, Dimitrios Rizos, Alfonso Gutiérrez-Adán

**Affiliations:** 1 Department of Animal Reproduction, INIA-CSIC, 28040 Madrid, Spain; 2 Mistral Fertility Clinics S.L, Clínica Tambre, Calle Tambre, 8, 28002 Madrid, Spain

**Keywords:** ART, Bovine, Sperm morphometry, Sperm selection, Thermotaxis

## Abstract

**Background:**

Sperm migration by thermotaxis is a guidance mechanism that operates along the oviduct and it has proved to be a valid method for selecting spermatozoa with low DNA fragmentation (SDF) in mice, humans, and stallions. This study aimed to analyse if bull spermatozoa could be selected by thermotaxis and to assess their quality in terms of SDF as well as determine the presence of a specific sperm subpopulation based on sperm morphometry and assess their fertilizing capacity by ICSI.

**Methods:**

We used frozen-thawed sperm from 6 bulls and sperm selection by thermotaxis was performed with TALP medium supplemented with 25 mmol/L of HEPES and 5 mmol/L of caffeine. In these conditions, sperm selection was achieved, obtaining a net thermotaxis of 3.6%. Subsequently, we analysed the SDF of the migrated and not-migrated spermatozoa using the neutral COMET assay, and we evaluated the size of the sperm head using Hemacolor® staining with Motic Images Plus 3 software. Additionally, migrated and not-migrated spermatozoa by thermotaxis were used to fertilize bovine in vitro matured (IVM) oocytes by ICSI, a very inefficient procedure in cattle that is only successful when the oocyte is artificially activated.

**Results:**

The results showed lower SDF (χ², *P* < 0.001, 13.3% reduction, *n* = 8) and lower head size parameters (length and width, *P* < 0.01; and perimeter and area, *P* < 0.001; *n* = 4) in those spermatozoa migrated in comparison to those not-migrated. The distribution of sperm subpopulations structure varied between groups, highlighting cluster 2, characterized by spermatozoa with small head size, and high ellipticity and elongated heads, as the most abundant in the thermotaxis migrated group. When performed ICSI (without oocyte artificial activation) with the thermotactic sperm, the blastocyst rate was 32.2% ± 9.3% in the group microinjected with the thermotactic spermatozoa vs. 8.3% ± 7.8% in the group of not-migrated sperm (χ², *P* < 0.05).

**Conclusion:**

Our results showed that bull sperm selection by thermotaxis has a much higher DNA integrity, small and elongated head size parameters, and different sperm subpopulation structure than the not-selected spermatozoa. Additionally, we evidenced that thermotactic spermatozoa improve ICSI success rates.

**Supplementary Information:**

The online version contains supplementary material available at 10.1186/s40104-022-00810-3.

## Background

It has been shown that, in mammals, from the millions of spermatozoa ejaculated, only tens to hundreds reach the site of fertilization at the ampulla [1]. Presumably, this selected group of spermatozoa have better characteristics for supporting fertility and embryo development [2]. The guidance of the spermatozoa through the female genital tract has been proposed to be a physiological phenomenon to help them reach the oocyte in vivo [3]. Different guidance mechanisms have been described in vitro which are chemotaxis [4], rheotaxis [5] and thermotaxis [6]. Sperm thermotaxis is defined as the ability of the spermatozoa to move in a temperature gradient from the lower to the higher [6], and it is present in different species such as rabbits, humans [6], mice [7], horses [8] and also in bull [9]. However, in bull, it has been evaluated the ability of the spermatozoa to sense the gradient but not the quality of the spermatozoa migrated [9]. Considering the presence of this phenomenon in different species, this guidance mechanism could be understood as a phenomenon conserved among species.

In mice, humans and horses, the recovery of the spermatozoa migrated by thermotaxis has shown lower levels of DNA fragmentation than the not-selected ones; while in mice, intracytoplasmic sperm injection (ICSI) with spermatozoa selected by thermotaxis increased the production of blastocysts with higher quality and percentage of live births [7]. Sperm DNA fragmentation (SDF) has been linked to poor rates of embryonic development and conception and increased number of miscarriages, morbidity in the offspring and even childhood cancer [10]. Besides, in mice, DNA fragmentation has also been linked to long-term multigenerational consequences and premature ageing, such as aberrant growth, mesenchymal tumours, and abnormal behavior [11]. Moreover, we have reported that the use of DNA-damaged sperm reduced the rates of preimplantation embryo development and reduced the number of offspring [12]. In addition, during the passage of the sperm through the epididymis, the compaction of the chromatin is produced due to the change from histones to protamines, which reduces the size of the sperm head [13]. Therefore, a bigger sperm head area has been associated with deficiencies in chromatin compaction and higher abnormalities [14], which impairs embryo development in bovine. Chromatin compaction and thus, its integrity, could be indirectly evaluated by analyzing the sperm head morphology (size and shape) [15]. Taken all these together, efficient sperm selection mechanisms are needed to select viable and high-quality spermatozoa.

In bovine in vitro fertilization (IVF), the most common sperm selection technique is density gradient centrifugation (DGC). Using DGC, the IVF success rate in terms of the percentage of cleavage can reach up to 80% but embryo production remains between 30% and 40% [16, 17]. In contrast, ICSI is a very inefficient procedure in bovine that is only successful when the oocytes are artificially activated [18] yielding a low number of blastocysts (about 20%) [19, 20]. This inefficiency can be due to spermatozoa delayed or incomplete nucleus decondensation [21]. Even though IVF is widely used, the development of ICSI in bovine could be useful to decrease the generation interval, use of high valuable males and use semen samples with low sperm numbers, such as those obtained after sex-sorted [22]. Interestingly, in IVF it is necessary to use at least 10,000 spermatozoa per oocyte to have a good cleavage rate [23] suggesting that not all the spermatozoa are equally competent to fertilize the oocyte.

An approach to overcome the problems of ICSI in bovine would be to mimic some of the events that would occur in vivo during fertilization and that are bypassed with ICSI [24], such as the sperm selection by thermotaxis as it is a guidance mechanism known to operate in the oviduct [6]. It has been previously described in human, rabbit and mouse that the spermatozoa have to be capacitated to be able to sense the temperature gradient [6, 7]. Capacitation involves all those biochemical and structural changes that spermatozoa must undergo to be able to penetrate an oocyte [25]. Heparin is widely used for bull sperm capacitation in vitro [26]; however, other molecules have been used for sperm capacitation in vitro like caffeine [27, 28], among others [29–31].

Bovine sperm has been reported to sense a temperature gradient and move toward the warmer side [9]. However, analysis of the quality of the migrated spermatozoa, and its use in ICSI has not been performed yet. Consequently, this study aimed to evaluate the migration of frozen-thawed bull spermatozoa by thermotaxis with the methodology described by Pérez-Cerezales et al. [7] and establish the conditions of capacitation necessary for this system [7]. Besides, quality evaluation of the migrated spermatozoa was performed, in terms of SDF, head morphometry, and subpopulation structure, while their fertilizing competence through ICSI was assessed.

## Methods

### Experimental design

This study aimed to evaluate the ability of frozen-thawed bull spermatozoa to migrate by the thermotactic system developed by our group and to assess the quality of the spermatozoa recovered according to the DNA integrity, and size of the sperm head and developmental rates (cleavage rate and blastocyst yield) by ICSI. First, we examined whether or not bull spermatozoa were able to migrate during 1 h of selection in the thermotactic system with a temperature gradient from 36 ℃ to 39 ℃, using the FERT medium supplemented with 5 mmol/L of caffeine and 25 mmol/L of HEPES. Then, the sperm DNA fragmentation was evaluated using the neutral version of the comet assay and the size of the head of the spermatozoa selected was analysed using the Hemacolor® staining. Finally, the fertilizing competence of the thermotactic selected spermatozoa was tested in vitro by ICSI using in vitro matured oocytes (IVM), without activation.

### Sperm processing

Approval from an ethical committee to conduct this study was not required as all performed experiments were in vitro. Frozen-thawed seminal samples from 6 different Asturian Valley bulls with proven fertility were provided by the Regional Service of Agrifood Research and Development (SERIDA), Gijón, Spain. Animals were selected based on their artificial insemination (AI) outcomes using frozen samples being above 50% of the non-return rate (62% ± 9%, *n* = 6). Four to five straws (0.25 mL) of each bull (same ejaculate) were thawed at 37 ℃ in a water bath for 40 s. Motile spermatozoa were selected by BoviPure™ gradient (Nidacon Laboratories AB, Göthenborg, Sweden) centrifuged for 10 min at 290 × *g*. The resulted pellet was then resuspended in Boviwash solution (Nidacon Laboratories AB, Göthenborg, Sweden) and centrifuged for 5 min at 290 × *g*. Finally, the pellet was resuspended to a final concentration of 10 × 10^6^ spz/mL in FERT medium [Tyrode’s medium with 25 mmol/L bicarbonate, 22 mmol/L sodium lactate, 1 mmol/L sodium pyruvate, and 6 mg/mL fatty acid-free bovine serum albumin (BSA)] supplemented with 10 µg/mL of heparin or 5 mmol/L of caffeine and 25 mmol/L of HEPES.

### Sperm thermotaxis

Sperm thermotaxis was performed as previously described by Pérez-Cerezales et al. [7] with some modifications. Briefly, the medium used for bull sperm selection was FERT supplemented with 25 mmol/L of HEPES and 5 mmol/L of caffeine. The temperature gradient was set up from 36 ℃ to 39 ℃. The spermatozoa were loaded at a concentration of 10 × 10^6^ spz/mL in the drops at 36 ℃ (for migration by thermotaxis and for the temperature control) and the drops at 39 ℃ (for the inverted control and constant control temperature) and they were allowed to migrate for 1 h. After this time, migrated and not-migrated spermatozoa were recovered and processed for SDF, hemacolor staining analysis, and ICSI. The controls for random migration were the same as the previous study [7] but modified the temperature, therefore the non-gradient controls were at the same temperatures (36 ℃ to 36 ℃ and 39 ℃ to 39 ℃) and the inverted control was set from 39 ℃ to 36 ℃. The percentage of net thermotaxis was calculated as follows: [100 × (number of spermatozoa migrating within the temperature gradient (36 ℃ to 39 ℃) − number of spermatozoa migrating within the controls (selecting the control, which resulted in higher random migration)/number of spermatozoa loaded]. For the initial setting of the system, all controls were used. For the analysis of DNA fragmentation and ICSI, the inverted control was the only control settled as it was necessary to recover the maximum spermatozoa migrated by thermotaxis due to the low numbers recovered.

### DNA fragmentation analysis

DNA fragmentation was analysed employing the neutral version of the single cell gel electrophoresis assay (SCGE or Comet assay) as previously described with some modifications [7]. Briefly, the samples were pelleted by centrifugation (600 × *g*) and diluted to a maximum of 20 × 10^4^ spz/mL in 0.5% low melting point agarose in PBS. Because of the low numbers obtained in the thermotaxis assay, the samples of migrated spermatozoa were accumulated (pull of four capillaries) and used entirely. Immediately after dilution, 85 µL were placed on a slide previously coated with 1% agarose and covered with a 22 mm × 22 mm coverslip. The slides were then left at 4 ℃ for 15 min for agarose polymerization. Then, the lysis protocol used was the previously described by Ribas-Maynou specifically designed for bull sperm [32]. After lysis, the slides were washed for 30 min in neutral electrophoresis solution (90 mmol/L Tris, 90 mmol/L boric acid, and 2 mmol/L EDTA, pH 8.5) and then subjected to electrophoresis (25 V, 300 mA, for 10 min). After this, the slides were then washed in neutralization solution (0.4 mol/L Tris-HCl, pH 7.4) for 30 min, then washed in distilled water for 10 min and finally fixed in methanol for 3 min, air-dried, and stored until analysis. The samples were stained with 30 µL of ethidium bromide, covered with a 22 mm × 22 mm coverslip, and subsequently observed in a fluorescence microscope Nikon Optiphot-2 (Nikon, Tokyo, Japan). Comets were digitalized with a Nikon 5100 digital camera (Nikon, Tokyo, Japan) coupled to the microscope. From 150 to 200 comets were analysed per sample using the free software Casplab 1.2.3beta2 (CaspLab.com) [33].

### Sperm morphometry evaluation

To evaluate sperm head morphometry (length, width, perimeter and area of the head) the Hemacolor® Rapid staining (Merck, Madrid, Spain) was used for the spermatozoa in both migrated and not-migrated groups after thermotaxis. A total of 5 µL of the not-migrated group was deposited on a microscope slide, then smeared and left to dry at room temperature (RT). Because of the low number of spermatozoa in the migrated group, a 20-µL drop was partially smeared in this group and then air-dried at RT. Hemacolor staining was applied following kit recommendations. Slides were stained by immersion for 3 min in a fixing solution, 2 min in a colour reagent red containing Eosin Y and 2 min in a colour reagent blue containing azur B. A total of 32 slides were analysed (8 slides from each replicate: 4 slides per experimental group). In each slide, 25 spermatozoa with well-formed heads were measured using the Motic BA210 microscope equipped with a Moticam 3.0 MP CMOS Digital Camera. A picture of the spermatozoa was obtained using a 100× lens and the area of the head was measured with the Motic Images Advanced 3.2 software.

Based on the data obtained from the measurement of the sperm head, calculations were made for the parameters related to the shape of the structure: ellipticity (length/width), rugosity (4π × area/perimeter^2^), elongation [(length − width)/(length + width)] and regularity (π × length × width/area) [34]. Elongation and ellipticity values describe the size of the cell in terms of how long (the higher the value, the longer the cell), and how wide it is (value above 1 refers to an elliptical cell, and a value equal to 1 is indicative of round cells), respectively. The regularity describes more precisely the format within the variations of the ellipse. A value equal to 1 indicates the perfect ellipse. The rugosity characterizes the amorphous cells with a lower value, indicating a rougher surface of the head. This parameter can be positively correlated with head length, as well as being indicative of cell susceptibility to damage/rupture [35].

### Acrosomal exocytosis analysis

The acrosomal exocytosis evaluation was performed as previously described [36]. Slides were washed for 5 min twice in PBS and 50 µL of 15 µg/mL of fluorescein isothiocyanate-conjugated peanut agglutinin (FITC-PNA) (L7381, Merck) and 6.5 µg/mL Hoechst 33,342 in PBS were added and covered with a 24 × 24 cover slide. The slides were then incubated for 30 min at room temperature in a humid box. Subsequently, the slides were washed in distilled water for 10 min and mounted with Fluoromount^TM^ aqueous mounting medium (Sigma-Aldrich, St. Louis, MO, USA), sealed with nail polish and examined in a fluorescence microscope Nikon Optiphot-2 (Nikon, Tokyo, Japan). At least 200 spermatozoa from two slides per sample were analysed by blind counting using codified slides. Only completely reacted acrosomes were counted as reacted and spermatozoa showing partial staining in the acrosome were counted as unreacted.

### Multivariate procedures analysis

Multivariate procedures were performed to identify sperm subpopulations according to the morphometric sperm variables. First, the data was standardized to avoid any scaling effect. The principal component analysis (PCA) was used to reduce the dimensionality of the data, previously testing the adequacy of the data using Bartlett’s sphericity test and KMO (Kaiser-Meyer-Olkin) test. Then, the principal components with eigenvalue > 1 (Kaiser criterion) were selected to compute the varimax-rotated principal components, which will explain a high percentage of the total variance. Afterwards, the non-hierarchical clustering method k-means was used to classify the spermatozoa by morphology into a reduced number of subpopulations. The k-means method uses a predefined number of clusters which was selected using the minimum total within-cluster sum of squares. Sperm subpopulations were characterized in terms of their morphometric variables.

### Oocyte collection and in vitro maturation

Bovine ovaries were recovered from a local slaughterhouse, and immature cumulus-oocyte complexes (COCs) were obtained by aspirating follicles (2–8 mm) from the ovaries of mature heifers and cows. After selection, COCs with homogeneous cytoplasm and intact cumulus cells (grade I and II) were maturated in groups of 50 COCs per well in four-well dishes (Nunc, Roskilde, Denmark) containing 500 µL maturation medium (TCM-199), supplemented with 10% fetal calf serum (FCS) and 10 ng/mL epidermal growth factor (EGF). Oocytes were IVM for 24 h at 38.5 °C, with 5% CO_2_ in air with maximum humidity.

### In vitro fertilization (IVF)

As a control for the ICSI, we set a parallel standard IVF group using the IVM oocytes from the same batch and the spermatozoa selected by DGC. As previously described in sperm processing, frozen semen straws from a bull of proven fertility were thawed at 37 ℃ in a water bath for 40 s. Sperm selection was performed with Bovipure (Nidacon Laboratories AB, Göthenborg, Sweden), and the final sperm concentration was adjusted to 1 × 10^6^ spz/mL for fertilization. Gametes were co-incubated in 500 µL fertilization medium (Tyrode’s medium with 25 mmol/L bicarbonate, 22 mmol/L sodium lactate, 1 mmol/L sodium pyruvate, and 6 mg/mL fatty acid-free BSA) supplemented with 10 µg/mL heparin sodium salt (Calbiochem, San Diego, CA, USA) in a four-well dish, in groups of 50 COCs, for 18–20 h at 38.5 °C, 5% CO_2_ in air with maximum humidity.

### Intracytoplasmic sperm microinjection (ICSI)

ICSI was performed with Piezo-actuated micromanipulation adapted from mouse ICSI protocols, previously described [37] instead of conventional ICSI with a sharp injection needle. ICSI procedure was carried out on a 90-mm Petri dish, with a 50-µL drop of Holding medium (TCM 199, 40% Earle’s Salts (Gibco, 31150-022); 40% Hanks Salts with 25 mmol/L HEPES (Gibco, 22350-029); and 20% Fetal Calf Serum) for oocyte manipulation, a 10% PVP in PBS drop for injection needle wash; and several 20 µL drops of a mixture 1:5 of 10% PVP in PBS: Holding medium, covered by mineral oil. Bovine oocytes were injected in groups of 10, alternating among sperm groups. Sperm was kept in the same medium used in thermotaxis, at 38 °C, and 5 µL was placed in a fresh drop immediately before its use. Spermatozoon with proper motility was captured by suction with the injection needle and, after breaking the mid-piece of the flagellum by a piezo pulse to avoid any movement of the spermatozoon, they were moved to a manipulation drop, and injected into the oocyte cytoplasm after passing through the zona pellucida. Injected oocytes were recovered from the manipulation drop and placed in a culture medium for embryo development. Furthermore, a control group was settled in which the oocytes were injected with the needle without spermatozoa (sham group).

Injected and control oocytes were cultured in groups of 20–25 zygotes in 25 µL droplets of synthetic oviduct fluid (SOF) [38] with 4.2 mmol/L sodium lactate, 0.73 mmol/L sodium pyruvate, 30 µL/mL BME amino acids, 10 µL/mL MEM amino acids, 1 µg/mL phenol-red and 5% FCS (F2442, Sigma). Approximately 18–20 h post-insemination (hpi), presumptive zygotes from the control group were denuded of cumulus cells by vortexing for 3 min and then cultured in groups of 20–25 in 25 µL droplets of SOF covered with mineral oil at 38.5 °C, 5% CO_2_, 5% O_2_ and 90% N_2_. Cleavage rate was recorded on day 2 (48 hpi) and blastocyst yield on day 7 post-insemination. A total of 8 replicates were used to assess embryo development after ICSI.

### Statistical analysis

Data were analyzed by descriptive statistics based on the mean ± standard deviation calculated for each of the variables. Differences among treatments were analyzed using one-way ANOVA, and Post-hoc analysis to identify differences between groups was performed using Tukey test for parametric analysis or Kruskal-Wallis’s test for non-parametric analysis. A Chi-square test (χ²) was used to analyze the differences between samples. A Z-test was used for the analysis of the percentage of acrosome-reacted spermatozoa and percentages of spermatozoa subpopulations. Differences were considered significant when *P* < 0.05. One-way ANOVA was performed to analyze the differences between sperm subpopulations, followed by pairwise *t*-tests. Statistical significance was considered as *P* < 0.05. All data were analyzed using R (v 4.1.3).

## Results

### Sperm migration by thermotaxis using heparin vs. caffeine as capacitating molecules

Because sperm capacitation is a prerequisite to respond to thermotaxis, we first used heparin for inducing sperm capacitation as it is widely added in the capacitation media [26]. The medium used was FERT supplemented with 25 mmol/L of HEPES with heparin at a concentration of 10 µg/mL, which is the standard protocol used for bull sperm capacitation. In these conditions, we observed that heparin produced a strong head-to-head agglutination, as previously reported [26] which affected sperm motility and prevented sperm from migrating in response to thermotaxis. Then, we analyzed whether caffeine, which has been previously described as a capacitating agent in frozen-thawed bull spermatozoa [27], could promote capacitation without increasing sperm agglutination. We supplemented the FERT medium with 25 mmol/L of HEPES and 5 mmol/L of caffeine for sperm migration by thermotaxis and we found that this concentration, which has been reported that induced capacitation in bovine spermatozoa [39], avoided sperm head-to-head agglutination, therefore, it was the medium of choice.

**Fig. 1:**
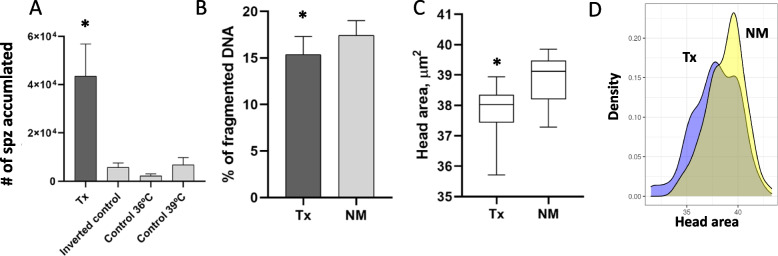
Number, DNA fragmentation, and head size of the spermatozoa selected by thermotaxis. **A** Number of spermatozoa selected by thermotaxis with the medium supplemented with 5 mmol/L of caffeine in the thermotactic migration unit, thermotaxis (Tx) group (36 to 39 ℃), the inverted control (39 to 36 ℃) and the constant control temperatures (36 to 36 ℃ and 39 to 39 ℃). The asterisk indicates significant differences between groups (Kruskal-Wallis, *P* < 0.05). The initial number of spermatozoa loaded in the thermotaxis system was 10 × 10^6^ spz/mL per separation unit, *n* = 6. **B** Percentage of DNA fragmentation in the spermatozoa selected by thermotaxis (Tx) and the not-migrated spermatozoa (NM), *n =* 8. Asterisk indicates significant differences between groups. **C** Area of the head size from spermatozoa. Number of spermatozoa migrated by thermotaxis (Tx) and not-migrated (NM) (4 repetitions; *n* = 400 spermatozoa analyzed for repetition). χ², *P* < 0.01. **D** Representation of the distribution of the head area value of spermatozoa migrated by thermotaxis (Tx) and not-migrated (NM)

When thermotaxis was performed under these conditions, we obtained a higher number of sperm migrated in the thermotactic unit (from 36 ℃ to 39 ℃) than the rest of the controls (*P* < 0.006, *n* = 6) (Fig. 1 A). This means that, a higher number of spermatozoa migrated by sensing the gradient, from the lower temperature (36 ℃) to the higher temperature (39 ℃), than the random migration obtained in the controls of constant temperature (at 36 ℃ and 39 ℃) and the inverted control (migration from 39 ℃ to 36 ℃). The average of the total number of cells migrated by thermotaxis was 4.3 × 10^4^ spz and the percentage of net thermotaxis obtained with this medium was 3.6% (*n* = 6). We did not find differences between the number of sperm that migrated randomly in the three controls established (controls of constant temperatures at 36 ℃ and 39 ℃ and the inverted control from 39 ℃ to 36 ℃).

### Effect of sperm thermotaxis on DNA integrity

We next examined, by neutral COMET assay, if the spermatozoa migrated by thermotaxis had higher DNA integrity. We obtained a lower SDF level in the spermatozoa migrated by thermotaxis (from 36 to 39 ℃) in comparison to the spermatozoa not-migrated, that remained in the thermotactic separation unit (the remaining spermatozoa in the drop at 36 ℃ that was not able to migrate to the drop at 39 ℃) (Fig. 1B) (*P* < 0.001, χ², *n* = 8). The obtained percentage of reduction in the DNA fragmentation was 13.3% between groups.

### Sperm head morphometry differences

The results of the comparison of the morphometric dimensions of sperm heads from spermatozoa migrated or not migrated by thermotaxis are summarized in Table 1. A total of 400 properly digitalized spermatozoa were analyzed (4 replicates). Kruskal-Wallis one-way analysis of variance on ranks showed a significant effect of migration by thermotaxis on sperm head morphometry. The four sperm-head parameters of size were significantly different; and of the shape parameters, only rugosity was different (Table 1; Fig. S1). Sperm that responded to thermotaxis had a smaller head size than sperm that do not respond (Fig. 1C,D). To eliminate the possibility that the sperm that migrate by thermotaxis have undergone the acrosome reaction and have therefore smaller head area, we analyzed the presence of the acrosome employing the fluorescent probes PNA-FITC [36] and found that in both cases the percentage that had completely lost the acrosome were similar (1.25% vs. 3.75% for not-migrated and migrated respectively).

**Table 1:** Mean values (± SD) of each morphometric parameter corresponding to bull sperm migrated or not migrated by thermotaxis

Item	Tx	NM
Length, µm	9.14 ± 0.38	9.24 ± 0.36^**^
Width, µm	4.17 ± 0.22	4.24 ± 0.23^**^
Perimeter, µm	32.45 ± 1.79	34.15 ± 2.03^***^
Area, µm^2^	37.83 ± 2.20	38.80 ± 1.82^***^
Ellipticity, L/W	2.20 ± 0.15	2.19 ± 0.15
Rugosity, 4πA/P^2^	0.46 ± 0.06	0.42 ± 0.05
Elongation, (L-W)/(L + W)	0.37 ± 0.03	0.37 ± 0.03
Regularity, πLW/4A	0.79 ± 0.07	0.79 ± 0.06

*SD* Standard deviation. Significant differences between Tx and NM by Kruskal-Wallis one-way analysis of variance on ranks, ^**^P < 0.01, ^***^P < 0.001

Interestingly, the head area distribution in thermotactive-responsive sperm had two peaks while the not-migrated group only had one (Fig. 1D). The same effect was observed in other parameters measured such as length, width, and ellipticity, suggesting that within the population of spermatozoa that migrate by thermotaxis, there may also be two subpopulations, one that could correspond to the once migrated correctly because responded to thermotaxis (a population that is different from non-migrated sperm) (Fig. 1D), while the other (population with a peak similar to the control) could be sperm that have migrated by chance, as it happens with the spermatozoa that migrate by chance in the control groups (Fig. 1 A).

### Sperm head subpopulation differences

**Fig. 2:**
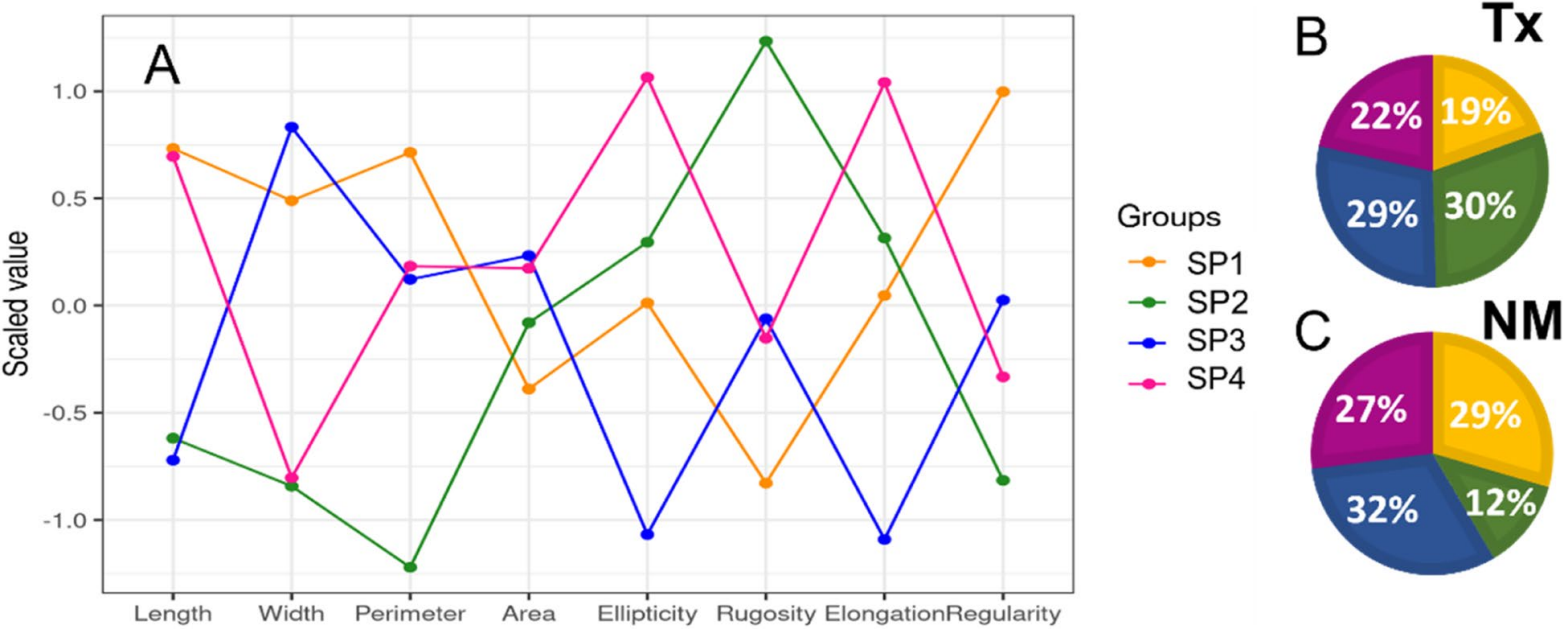
Subpopulation distribution of bull sperm selected by thermotaxis. **A** Scaled mean values of the 8 morphometric variables in the four sperm subpopulations. **B** Proportion of spermatozoa belonging to each subpopulation in the spermatozoa migrated by thermotaxis, and (**C**) in the not-migrated spermatozoa

The principal component (PC) analysis of the sperm head morphometry data from both spermatozoa migrated and not-migrated by thermotaxis, produced three PCs, explaining 89.1% of the variance (Table S1). PC1 was represented by length, width, ellipticity, and elongation components; PC2 was represented by perimeter and rugosity, and PC3 was represented by area and regularity. The analysis of subpopulations revealed four well‑defined groupings (Fig. S2). In Fig. 2 and Table S2, we can see the mean values of the morphometric parameter corresponding to the 4 subpopulations (SP) and the significant differences between SPs (Fig. S3). The characteristics of SP1 showed the highest PC2, SP2 showed the lowest PC2, SP3 showed the lowest PC1 and SP4 showed the highest PC1.

The spermatozoa from SP1 have high length, width, perimeter and regularity values, and this SP1 is reduced in the thermotactic group (Fig. 2). SP2 has low length, width, perimeter and regularity, but has high ellipticity and elongation, and higher rugosity value (poorly amorphous and rough cells). This subpopulation is the one that increases the most in the thermotactic group (Fig. 2). No differences were found for SP3 (higher width and lower elongation and regularity) and SP4 (higher length and higher ellipticity and elongation values). The results indicate that the spermatozoa that have migrated by thermotaxis have small length, width, and perimeter but are elongated in an elliptical shape and with very little roughness in their membrane, which corresponds to the SP2.

### ICSI with migrated spermatozoa

**Fig. 3:**
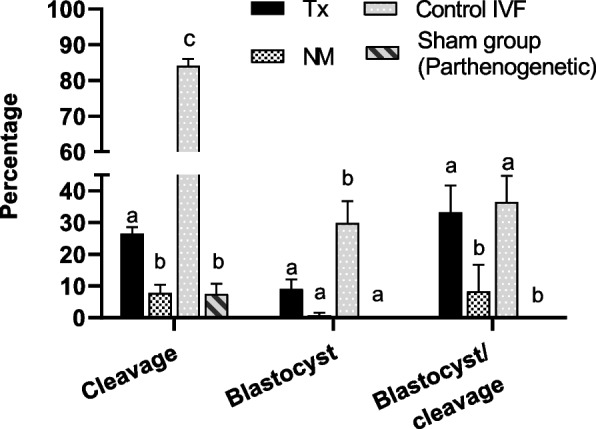
Rate of cleavage and blastocyst production. Percentage of cleavage and blastocyst production of the embryos obtained by ICSI using the spermatozoa migrated by thermotaxis (Tx) (*n* = 8, *P* < 0.01 for cleavage), not-migrated (NM) by thermotaxis (*n* = 8), obtained by conventional IVF (*n* = 6) and oocytes microinjected without sperm (Parthenogenetic) (*n* = 4). Different letters above error bars (i.e. ±SEM) indicate significant differences (*P* < 0.001) among groups (Tukey’s posthoc test)

The results obtained after ICSI, employing IVM oocytes with sperm selected by thermotaxis showed a significant higher cleavage rate in comparison to those microinjected with not-migrated spermatozoa and also with the sham group (oocytes microinjected without sperm (26.2% ± 1.7% vs. 9.3% ± 2.7% vs. 7.2% ± 3.1% respectively, *P* < 0.001) (Fig. 3). When looking at the blastocyst production (percentage of blastocyst out of the cleaved embryos) a significantly higher production was obtained when it was used migrated spermatozoa compared with those oocytes microinjected with the not-migrated sperm and the sham group (considered a parthenogenetic division) (32.2% ± 9.3% vs. 8.3% ± 7.8% and 0 respectively, *P* < 0.05). The IVF control (IVM oocytes and spermatozoa selected only by DGC) gave a cleavage rate of 84.1% ± 1.9% and a blastocyst yield of 35.5% ± 8.3% demonstrating that the oocytes were properly matured and the semen sample was adequate.

## Discussion

This study aimed to evaluate bull sperm selection by thermotaxis and the quality of the spermatozoa migrated according to their DNA fragmentation, sperm head morphometry, and their ability to fertilize IVM oocytes through ICSI. Here, we report the successful migration by thermotaxis of frozen-thawed bull sperm using the system previously developed by Pérez-Cerezales et al. [7]. Furthermore, to our knowledge, this is the first time that the quality of bull sperm migrated by thermotaxis is evaluated showing higher DNA integrity and lower sperm head size. Moreover, when used for ICSI (without oocyte activation) we were able to obtain a higher cleavage and blastocyst formation rates compared with the not-migrated group.

We applied here the system developed by Pérez-Cerezales et al. [7] as it can recover mice and human spermatozoa with high quality and in the case of mice, improve ICSI outcomes. Consequently, in this study, we adapted the temperature of sperm selection for bulls with respect to mice, humans and horses [7, 8] as the gradient established here went from 36 to 39 ℃ instead of 35 to 38 ℃. This change was made taking into consideration the normal core body temperature of a healthy cow, and the temperature of incubation in bovine IVF which is 38.5 ℃, therefore, this gradient would be the most suitable for bull sperm selection. The medium used in this study for migration by thermotaxis was the FERT, supplemented with 5 mmol/L of caffeine instead of heparin to avoid sperm head-to-head agglutination. In these conditions, migration by thermotaxis was achieved in bull sperm, obtaining a net thermotaxis of 3.6% which is higher than that obtained for stallion (1.1%), mouse (0.5%) and human (0.8%) using the same sperm selection system [7, 8]. The spermatozoa have to be capacitated to migrate by thermotaxis [6] and under capacitating conditions, we know that only about 10% of them are capacitated at a given point [40]. Therefore, it could be expected that the percentage of spermatozoa able to migrate by thermotaxis would be lower than 10%, as previously reported for different species [7, 8] and also confirmed with current results. Nevertheless, the net thermotaxis was higher for bull sperm in comparison to the species aforementioned, which could be due to the adequation of the capacitation medium according to the species-specific necessities or the origin of the semen sample used, as frozen samples (like the ones used for horse) gave a higher migration percentage than the fresh (mouse and human) [7, 8]. Should be taken into consideration that the frozen-thawed sperm show capacitation-like events due to the procedure of freezing and thawing itself, known as cryocapacitation [41] and, as a consequence of this, a certain degree of capacitation is obtained right after thawing. This is the reason why the thermotactic assay was performed immediately after DGC.

Sperm selected by thermotaxis has lower DNA fragmentation index than not-selected sperm. DNA fragmentation is one of the parameters whose analysis has gained great importance for the prediction of ART success [42]. Sperm DNA damage induces fragmentation of chromosomes and segregation errors leading to mosaicism of embryos [43] compromising embryonic development [44]. In this study, we found a reduction of 13.3% of the levels of DNA fragmentation in the sperm migrated by thermotaxis in comparison to the control confirming that we could select, with this methodology, a sperm subpopulation with higher quality in terms of DNA integrity as previously reported in other species [7, 8]. Taking into account that the conventional sperm selection techniques (swim-up and DGC) have not been able to demonstrate the selection of spermatozoa with lower SDF [45], the results reported here suggest that thermotaxis could be a useful sperm selection technique to recover the sperm subpopulation with better quality in terms of DNA integrity.

Another sperm quality parameter analyzed was sperm head morphology as it has been linked to the sperm nuclear compaction and chromatin integrity [15]. A bigger sperm head area has been associated with deficiencies in protamine compaction and higher abnormalities [14], while thinner sperm nuclei are related to higher fertilization rates in mice [14, 46]. Also, differences in sperm head length have been directly related to conception rates in other species. For example, an increase in the coefficient of variation of the sperm head length in bulls and stallions has been related to a reduction in fertility [47, 48]. Also, in red deer and ram, it has been reported that males with high fertility rates have ejaculates with a larger proportion of spermatozoa having small and elongated heads [49, 50]. In agreement with these, our results demonstrated that spermatozoa migrated by thermotaxis had a smaller sperm head size and higher elongation and ellipticity values in comparison to the not-migrated spermatozoa. Thus, the reduction of the head size and elongation of the spermatozoa migrated by thermotaxis could be related to the selection of spermatozoa with higher quality.

Finally, to test the quality of the bull spermatozoa migrated by thermotaxis, we performed ICSI, which is an inefficient procedure in bovine that is only successful when the oocyte is artificially activated [18]. We reported here that, without oocyte artificial activation, the percentage of cleavage rate and blastocyst yield were higher for those oocytes microinjected with spermatozoa selected by thermotaxis vs. those not-selected, as well as previously reported for mice [7]. Some of the causes of the inefficiency of ICSI in bovine are failure of the oocyte activation after microinjection and defective sperm head decondensation [19, 21, 51]. These results suggest that thermotaxis might have been able to select spermatozoa with the ability to activate the oocyte or decondense the sperm head properly after cytoplasmic injection. We hypothesized that spermatozoa selected by thermotaxis could be properly capacitated and this could be the reason why oocyte activation was not necessary to achieve similar or even higher embryo production rates in comparison to other studies that used ICSI with oocyte activation [19, 20, 51]. These results are prominent for future studies to optimize the current methodology for bull sperm selection by thermotaxis, which in combination with ICSI and oocyte activation might increase the success of ICSI in this species.

## Conclusion

Our data showed that bull sperm selection by thermotaxis was achieved using frozen-thawed semen and that the population selected had higher DNA integrity and lower sperm head size that the not-selected spermatozoa. Besides, when using this population to perform ICSI, higher cleavage and blastocyst formation rates were obtained in comparison to the not-migrated spermatozoa. As far as we know, this is the first report showing the increased quality of bull sperm migrated by thermotaxis and its successful use for ICSI, which could make sperm selection by thermotaxis an advantageous technique to improve ICSI outcomes in bovine.

### Supplementary Information

**Additional file 1:** **Table S1.** Eigenvalues of each parameter in the three PCs for bull sperm head morphometry found in sperm migrated or not migrated by thermotaxis.

**Additional file 2:** **Table S2.** Mean values (± S.D.) of each morphometric parameter corresponding to different SPs from bull sperm head morphometry found in sperm migrated or not migrated by thermotaxis.

**Additional file 3:** **Fig. S1.** Density plots showing the distribution of the 8 morphometric variables in both migrated and not-migrated spermatozoa after thermotaxis.

**Additional file 4:** **Fig. S2.** Distribution of sperm subpopulations according to their PC values.

**Additional file 5:** **Fig. S3.** Boxplots showing the distribution of the morphometric variables of the four subpopulations along with mean comparison *P*-values obtained by the t-test.

## Data Availability

Data sharing is not applicable to this article as no datasets were generated or analysed during the current study.

## Abbreviations

AI: Artificial insemination
BSA: Bovine serum albumin
COCs: Cumulus-oocyte complex
DGC: Density gradient centrifugation
EGF: Epidermal growth factor
FCS: Fetal calf serum
HS: Hemacolor staining
ICSI: Intracytoplasmic sperm injection
IVF: In vitro fertilization
IVM: In vitro matured
NM: Not-migrated
PCA: Principal component analysis
SDF: Sperm DNA fragmentation
Tx: Thermotaxis
