# Supplementary material for: Bull spermatozoa selected by thermotaxis exhibit high DNA integrity, specific head morphometry, and improve ICSI outcome
Source: J Anim Sci Biotechnol. 2023 Jan 11;14:11. doi: 10.1186/s40104-022-00810-3 (PMC9832681; doi:10.1186/s40104-022-00810-3)
Supplement: Supplementary file 1 — Additional file 1: Table S1. Eigenvalues of each parameter in the three PCs for bull sperm head morphometry found in sperm migrated or not migrated by thermotaxis. [file 40104_2022_810_MOESM1_ESM.docx]

**Table S1** Eigenvalues of each parameter in the three PCs for bull sperm head morphometry found in sperm migrated or not migrated by thermotaxis

| **Item** | **PC1** | **PC2** | **PC3** |
| --- | --- | --- | --- |
| Length | 0.612 | 0.604 |  |
| Width | -0.801 | 0.459 |  |
| Perimeter | -0.108 | 0.923 | 0.164 |
| Area |  | 0.124 | 0.948 |
| Ellipticity | 0.999 |  |  |
| Rugosity |  | -0.861 | 0.242 |
| Elongation | 1 |  |  |
| Regularity | -0.183 | 0.529 | -0.766 |
| Explained variation, % | 38.4 | 30.8 | 19.9 |

PCs: principal components
