# Supplementary material for: Bull spermatozoa selected by thermotaxis exhibit high DNA integrity, specific head morphometry, and improve ICSI outcome
Source: J Anim Sci Biotechnol. 2023 Jan 11;14:11. doi: 10.1186/s40104-022-00810-3 (PMC9832681; doi:10.1186/s40104-022-00810-3)
Supplement: Supplementary file 2 — Additional file 2: Table S2. Mean values (± S.D.) of each morphometric parameter corresponding to different SPs from bull sperm head morphometry found in sperm migrated or not migrated by thermotaxis. [file 40104_2022_810_MOESM2_ESM.docx]

**Table S2** Mean values (± SD) of each morphometric parameter corresponding to different SPs from bull sperm head morphometry found in sperm migrated or not migrated by thermotaxis

| **Item** | **SP1** | **SP2** | **SP3** | | | **SP4** |
| --- | --- | --- | --- | --- | --- | --- |
| Length | 9.46 ± 0.24^a^ | 8.96 ± 0.28^b^ | | 8.92 ± 0.26 ^b^ | 9.45 ± 0.30^a^ | |
| Width | 4.31 ± 0.13^a^ | 4.01 ± 0.17^b^ | | 4.39 ± 0.16^c^ | 4.02 ± 0.13^b^ | |
| Perimeter | 34.79 ± 1.77^a^ | 30.74 ± 1.35^b^ | | 33.55 ± 1.63^c^ | 33.68 ± 1.40^c^ | |
| Area | 37.51 ± 1.97^a^ | 38.15 ± 2.35^b^ | | 38.80 ± 1.94^b^ | 38.67 ± 1.83^b^ | |
| Ellipticity | 2.19 ± 0.08^a^ | 2.24 ± 0.1^b^ | | 2.03 ± 0.08^c^ | 2.35 ± 0.10^d^ | |
| Rugosity | 0.39 ± 0.04^a^ | 0.51 ± 0.05^b^ | | 0.44 ± 0.04^c^ | 0.43 ± 0.04^c^ | |
| Elongation | 0.37 ± 0.01^a^ | 0.38 ± 0.02^b^ | | 0.34 ± 0.02^c^ | 0.40 ± 0.02^d^ | |
| Regularity | 0.86 ± 0.05^a^ | 0.74 ± 0.05^b^ | | 0.79 ± 0.05^c^ | 0.77 ± 0.04^d^ | |

SPs: subpopulations; SD: standard deviation. Values with different superscripts (a, b, c, d) in the same row were significantly different (Kruskal-Wallis one-way analysis of variance on ranks, *P* < 0.01)
