# Supplementary material for: Bull spermatozoa selected by thermotaxis exhibit high DNA integrity, specific head morphometry, and improve ICSI outcome
Source: J Anim Sci Biotechnol. 2023 Jan 11;14:11. doi: 10.1186/s40104-022-00810-3 (PMC9832681; doi:10.1186/s40104-022-00810-3)
Supplement: Supplementary file 3 — Additional file 3: Fig. S1. Density plots showing the distribution of the 8 morphometric variables in both migrated and not-migrated spermatozoa after thermotaxis. [file 40104_2022_810_MOESM3_ESM.docx]

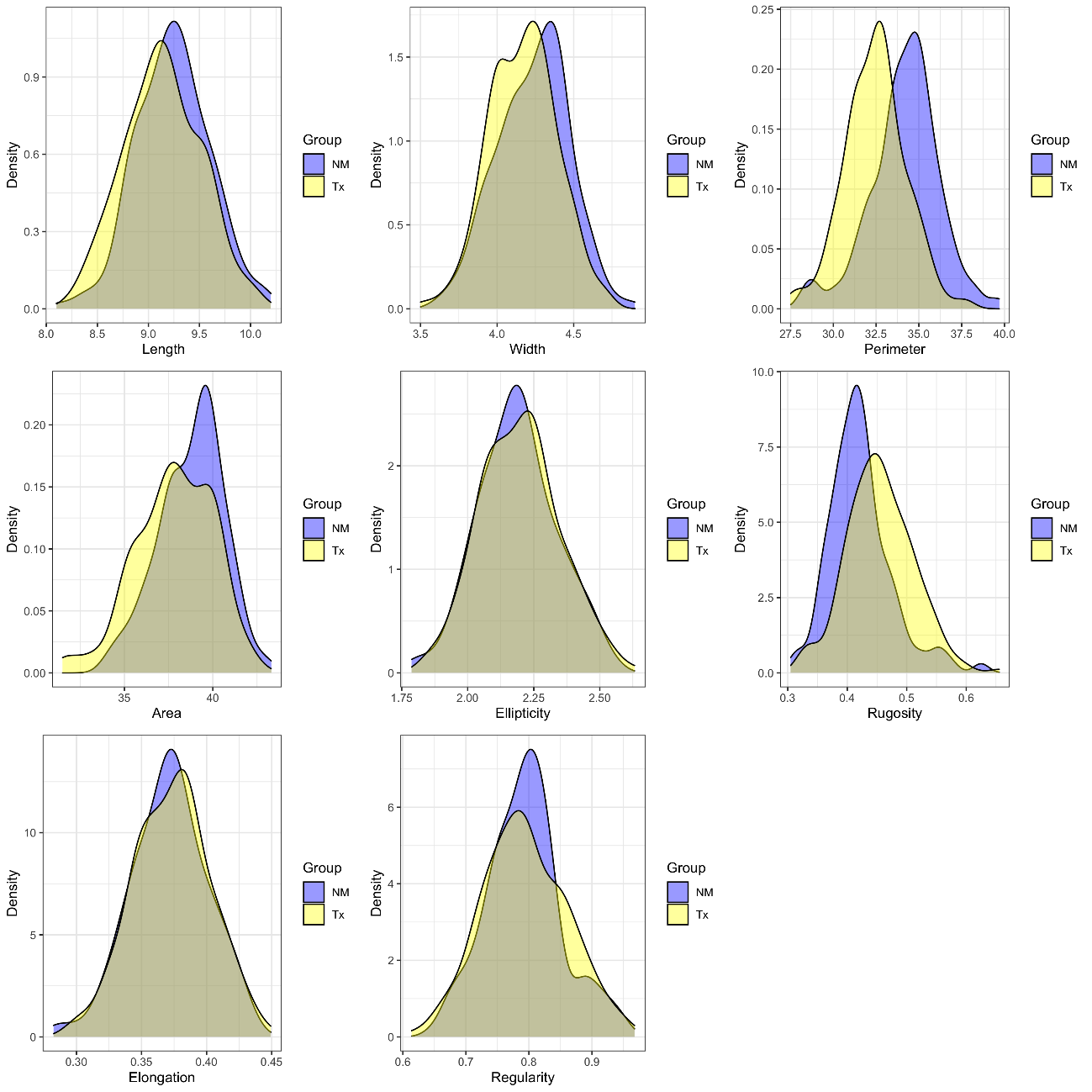


**Fig. S1.** Density plots showing the distribution of the 8 morphometric variables in both migrated and not-migrated spermatozoa after thermotaxis.
