# Supplementary figures and images for: Bull spermatozoa selected by thermotaxis exhibit high DNA integrity, specific head morphometry, and improve ICSI outcome
Source: J Anim Sci Biotechnol. 2023 Jan 11;14:11. doi: 10.1186/s40104-022-00810-3 (PMC9832681; doi:10.1186/s40104-022-00810-3)

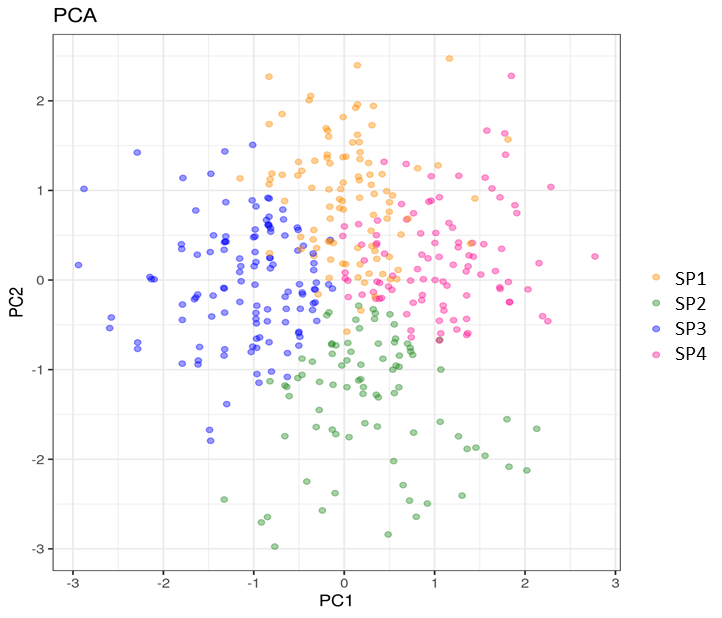


**Fig. S2**. Distribution of sperm subpopulations according to their PC values.

Supplement: Supplementary file 4 — Additional file 4: Fig. S2. Distribution of sperm subpopulations according to their PC values. [file 40104_2022_810_MOESM4_ESM.docx]
