# Supplementary material for: Bull spermatozoa selected by thermotaxis exhibit high DNA integrity, specific head morphometry, and improve ICSI outcome
Source: J Anim Sci Biotechnol. 2023 Jan 11;14:11. doi: 10.1186/s40104-022-00810-3 (PMC9832681; doi:10.1186/s40104-022-00810-3)
Supplement: Supplementary file 5 — Additional file 5: Fig. S3. Boxplots showing the distribution of the morphometric variables of the four subpopulations along with mean comparison P-values obtained by the t-test. [file 40104_2022_810_MOESM5_ESM.docx]

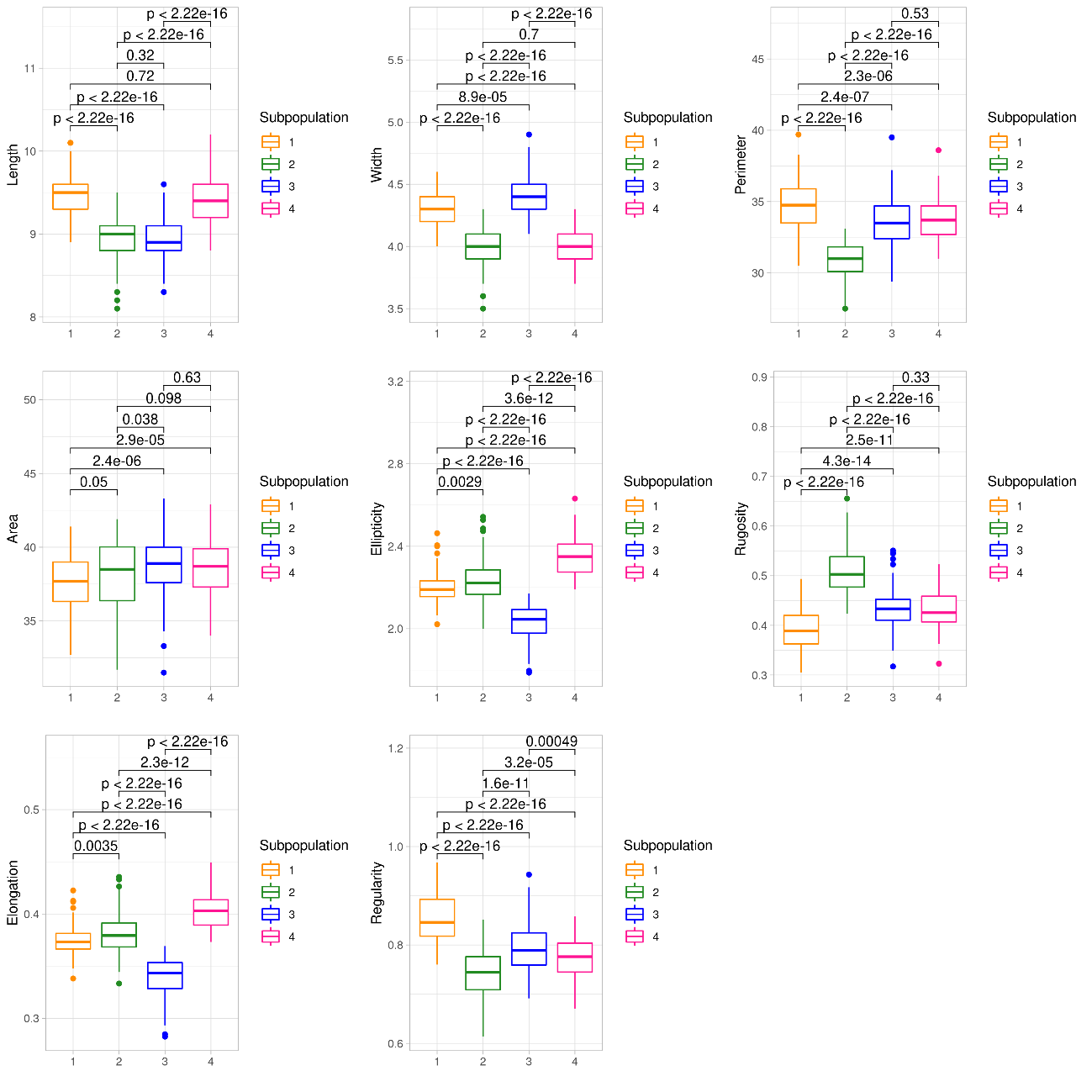


**Fig. S3**. Boxplots showing the distribution of the morphometric variables of the four subpopulations along with mean comparison *P*-values obtained by the t-test.
